# Exercise-induced seizures and lateral asymmetry in patients with temporal lobe epilepsy^☆^^☆☆^

**DOI:** 10.1016/j.ebcr.2013.12.004

**Published:** 2014-02-01

**Authors:** Jordan T. Kamel, Radwa A.B. Badawy, Mark J. Cook

**Affiliations:** St. Vincent's Hospital Melbourne, Department of Neurology & Neurological Research, Victoria, Australia

**Keywords:** Cortical localization, Reflex epilepsy, Exercise, Seizure triggers

## Abstract

**Objective:**

The objective of this case report is to better characterize the clinical features and potential pathophysiological mechanisms of exercise-induced seizures.

**Methods:**

We report a case series of ten patients from a tertiary epilepsy center, where a clear history was obtained of physical exercise as a reproducible trigger for seizures.

**Results:**

The precipitating type of exercise was quite specific for each patient, and various forms of exercise are described including running, swimming, playing netball, dancing, cycling, weight lifting, and martial arts. The level of physical exertion also correlated with the likelihood of seizure occurrence. All ten patients had temporal lobe abnormalities, with nine of the ten patients having isolated temporal lobe epilepsies, as supported by seizure semiology, EEG recordings, and both structural and functional imaging. Nine of the ten patients had seizures that were lateralized to the left (dominant) hemisphere. Five patients underwent surgical resection, with no successful long-term postoperative outcomes.

**Conclusions:**

Exercise may be an underrecognized form of reflex epilepsy, which tended to be refractory to both medical and surgical interventions in our patients. Almost all patients in our cohort had seizures localizing to the left temporal lobe. We discuss potential mechanisms by which exercise may precipitate seizures, and its relevance regarding our understanding of temporal lobe epilepsy and lateralization of seizures. Recognition of, as well as advice regarding avoidance of, known triggers forms an important part of management of these patients.

## 1 Introduction

The precipitation of seizures by exercise has been documented but has not been generally well described, with only a handful of cases reported in the current literature. These include patients with idiopathic generalized epilepsies (IGE) [1], [2], [3], as well as symptomatic focal epilepsies of frontal [4] and temporal [5] lobe origin. Although seemingly rare overall, exercise-induced seizures appear to be more commonly related to symptomatic focal epilepsies [6]. However, current literature more frequently supports a protective role of exercise against seizures, rather than an aggravating effect [6], [7].

There have been other case reports in earlier literature suggesting that seizures could be induced by voluntary movements, including running [8], [9]. However, many of these descriptions are more in keeping with a diagnosis of paroxysmal kinesigenic choreoathetosis [10], rather than a reflex epilepsy, especially given the preservation of consciousness in these cases, and an autosomal dominant inheritance pattern. There is a notable case by Falconer et al. of a patient with left frontal seizures that were precipitated by sudden movement of the contralateral leg [11]. Complete remission was achieved following resection of a cortical scar in the supplementary motor area.

We report a case series of ten patients from a tertiary epilepsy center, where a clear history was obtained of physical exercise as a reproducible trigger for seizures. Of note, all patients had seizures of temporal lobe origin, with all but one patient documented to have events lateralized to the left (dominant) side. We discuss possible mechanisms by which exercise may precipitate seizures and relevance regarding our understanding of temporal lobe epilepsy and lateralization of seizures, as well as clinical recommendations regarding exercise in patients with epilepsy.

## 2 Case series

### 2.1 Patient 1

**Fig. 1:**
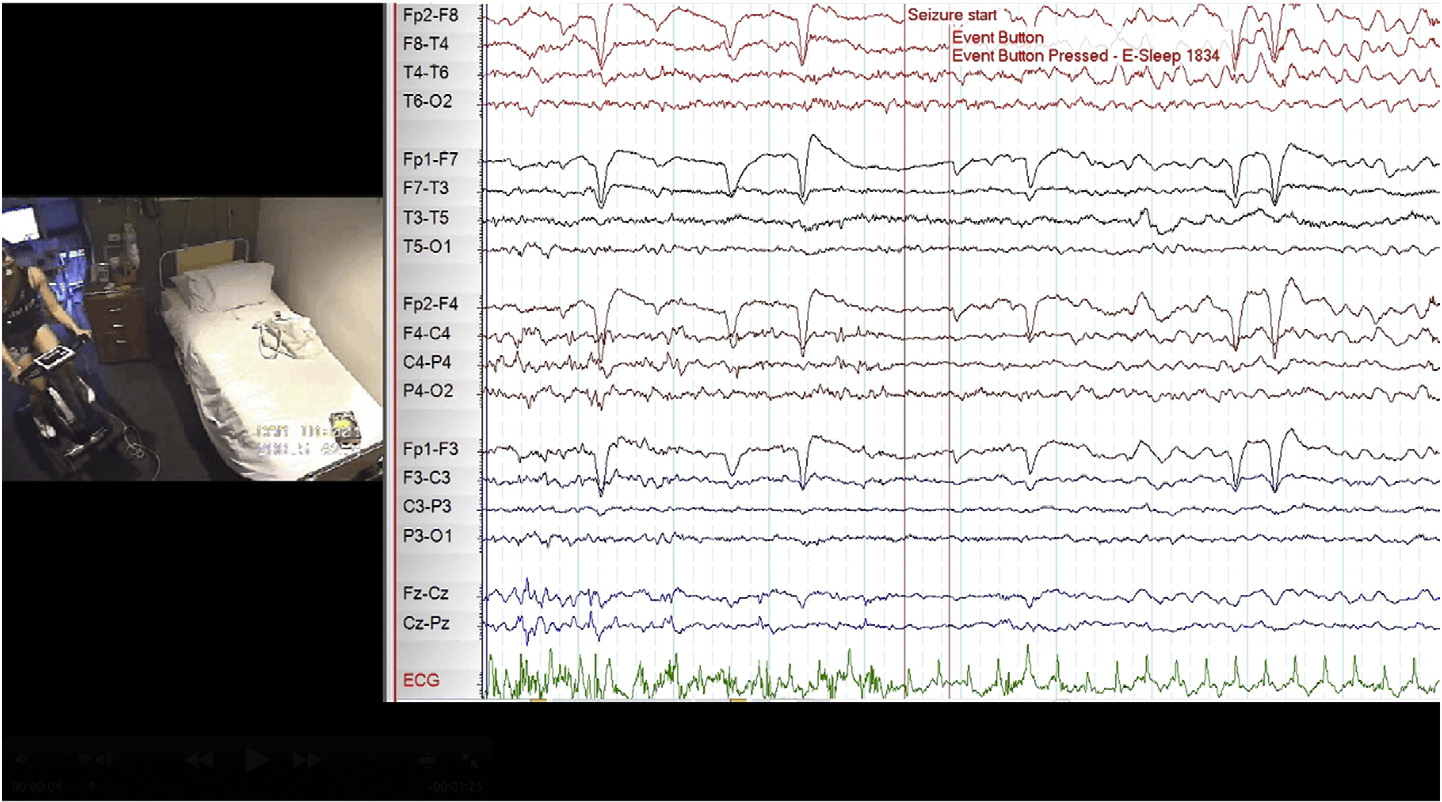
Exercise-induced seizure captured during video-EEG monitoring 5 min into cycling.

Patient 1 is a 28-year-old right-handed woman with a long history of complex partial seizures secondary to temporal lobe epilepsy (TLE). Attacks were often precipitated by vigorous exertion, particularly on the exercise bicycle at the gym. The severity of events would range from isolated auras of déjà vu and gustatory hallucination to loss of contact with manual and oral automatisms. Although seizures were not exclusive to exercise, the events were reliably induced with exertion. This was recorded during video-EEG monitoring (see Fig. 1 and the attached video online). As seizures were quite refractory to antiepileptic drugs (AEDs), and EEG, MRI, and PET scan all corresponded to right anterior temporal seizures, the patient underwent a right temporal lobectomy. Unfortunately, the postoperative outcome was not favorable, and she continued to have seizures including exercise-induced events. Seizure freedom was achieved, however, when lacosamide was added to her medication regime, which included topiramate and oxcarbazepine. This included seizure freedom during high levels of exercise.

### 2.2 Patient 2

Patient 2 is a 49-year-old right-handed man with focal seizures secondary to cortical dysplasia of the left superior temporal gyrus. Seizures would most frequently occur during exercise, particularly running, when most of his secondarily generalized seizures would occur. Seizures would only occur in the latter part of his runs, after several kilometers, when he was especially exerting himself. As the patient's seizures were refractory to four AEDs, with several other agents trialed in the past, surgery was discussed but not performed because of concerns regarding speech areas in the brain being affected. He stopped running entirely five years ago up to the present. This resulted in a marked reduction in seizures, with only one convulsive episode annually at the most and infrequent minor seizures. Although it was against medical advice, the patient started swimming to maintain some form of exercise. Interestingly, this did not precipitate any increase in seizures as was noted with his running.

### 2.3 Patient 3

Patient 3 is a 63-year-old right-handed man, formerly a professional footballer, who was referred to our epilepsy clinic with a history of left hippocampal sclerosis, which was surgically resected 15 years earlier. Although seizure frequency decreased as a result, the patient's epilepsy remained quite refractory to treatment with four AEDs. Although some seizures would occur from sleep, the patient identified physical exertion as another clear precipitant. This was fairly reproducible, and seizure control improved dramatically when he avoided any vigorous exercise. Gentle walking was not associated with any issues. Retrospectively, the patient felt that exercise had been a part of the pattern for years, even prior to his surgery. Interestingly, in addition to his typical seizures, exercise could induce a bout of severe depressive feelings. He also found that there was a family history of depressive symptoms provoked by exercise. On review by a neuropsychiatrist, it was felt that these latter symptoms actually represented part of a postictal psychosis, and some improvement was made with the introduction of olanzapine.

### 2.4 Patient 4

Patient 4 is a 48-year-old right-handed woman with temporal lobe epilepsy with bitemporal independent seizures documented on video-EEG monitoring. Events followed an encephalitic illness of unclear etiology several years prior and were resistant to polytherapy with four AEDs, with seizures occurring at least weekly. The patient noted that they were much more likely to occur while playing her weekly game of netball in the evenings.

### 2.5 Patient 5

Patient 5 is a 50-year-old right-handed woman with Rasmussen's encephalitis, with progressive speech disturbance and deafferent right hand, as well as focal seizures involving the right hand and face. Brain MRI showed marked focal atrophy of the left frontal and temporal lobes. Simple partial seizures were noted by the patient to occur predictably whenever she went line dancing. After a trial of low-dose clonazepam prior to dancing showed no significant improvement and resulted in excessive sedation, the patient was forced to cease this activity.

### 2.6 Patient 6

Patient 6 is a 74-year-old right-handed man with long-standing temporal lobe epilepsy with PET scan showing bitemporal hypometabolism and video-EEG monitoring showing bitemporal seizure origin but more frequent on the left. Seizures would more often than not occur while riding his bicycle, and despite being advised not to cycle, the patient continued to do so with several accidents as a result.

### 2.7 Patient 7

Patient 7 is a 44-year-old right-handed man with intractable complex partial seizures with frequent secondary generalization due to left hippocampal sclerosis. This was refractory to medical and surgical treatment. Major seizures occurred weekly, most often with exercise, particularly at the gym when lifting heavy weights. Thankfully, he would get a prolonged warning of rising epigastric sensation, which would prompt him to stop what he was doing and avoid injury. A small dose of clonazepam was trialed to be taken prior to physical exertion with some effect.

### 2.8 Patient 8

Patient 8 is a 27-year-old right-handed woman with long-standing focal seizures, which began during an admission with an encephalitic illness in childhood. Events were stereotyped, with nausea followed by right arm tingling and occasional secondary generalization. Symptoms were noted to be more frequent during intensive physical exertion, particularly during martial arts training. Brain MRI showed extensive left temporal lobe atrophy with involvement of mesial temporal structures, and PET imaging showed corresponding hypometabolism. Depth electrodes confirmed that seizures originated from the left hippocampus, and temporal lobectomy was performed. Unfortunately, there was no improvement in her symptoms, and she remains on five antiepileptic agents.

### 2.9 Patient 9

Patient 9 is a 31-year-old right-handed man with ten years of focal seizures that would rapidly generalize, with the majority of events noted to occur while cycling. Video-EEG monitoring revealed a left temporal focus, but MRI was unremarkable. Positron emission tomography evaluation showed hypometabolism in the left anterior and mesial temporal cortex, and the patient proceeded with temporal lobectomy. Similar to the previous patients in this case series, this provided little improvement in seizure control.

### 2.10 Patient 10

Patient 10 is a 45-year-old right-handed man with seizures that occurred soon after a diagnosis of a venous sinus thrombosis a few years earlier. The events consisted of a feeling of déjà vu and then global dysphasia with retained awareness. On rare occasions, they would secondarily generalize. The patient noted that his seizures were more likely to occur during intense aerobic exercise. This included both running and cycling, and his seizures would occur at the end of his training or in the first few seconds after stopping. He also noted that events could be induced even when only thinking about exercise. Routine EEG was unremarkable. Magnetic resonance imaging did not show any intracerebral injuries that could relate to the previous sinus thrombosis but did show mild volume reduction of the left anterior temporal lobe structures. Positron emission tomography showed corresponding left anteromesial temporal hypometabolism. Some control was achieved on a combination of lamotrigine and levetiracetam, but exercise and thinking about exercise would still provoke seizures.

## 3 Discussion

We have described ten patients with exercise-induced seizures. Table 1 gives a summary of these patients and their characteristics. There were no patients in this case series with generalized epilepsy, consistent with previous literature that states that exercise-induced seizures are more commonly related to focal epilepsy syndromes [6].

In general, the level of physical exertion correlated with the likelihood of seizure induction, with rigorous exertion more likely to precipitate an event than gentle exertion. For example, Patient 3 was affected by running but not by walking. The precipitating type of exercise could also be specific for each patient. For example, Patient 2 would reliably have a seizure while running, yet swimming at a similar level of exertion did not trigger any events. Various forms of exercise were described amongst our patients including running, swimming, playing netball, dancing, cycling, weight lifting, and martial arts.

As the entity of this reflex epilepsy is not well recognized, the mechanism by which exercise exerts its epileptogenic effect is even less well understood. It is unlikely that it simply relates to hyperventilation associated with exercise, firstly, as there was no correlation with hyperventilation as an activation procedure during EEG recordings, and secondly, as such hyperventilation induces seizures through changes in acid–base balance rather than hyperventilation associated with exercise, which is a physiological compensatory response.

Nine of the ten patients had clearly documented temporal lobe epilepsies, supported by seizure semiology, EEG recordings, and both structural and functional imaging. Only Patient 6 had evidence of extratemporal involvement, with frontal lobe atrophy, in addition to the temporal lobe involvement seen on MRI. Although the patients in our case series described seizures that were triggered by motor activities, Patient 6 was the only patient who had an abnormality involving the motor cortex itself.

There is ample evidence from multiple transcranial magnetic stimulation (TMS) studies that epilepsy is characterized by an increase in motor cortical excitability [12], [13]. This was noted in patients with both generalized and focal epilepsies, even when the seizure focus was distant from the motor area, suggesting a highly interconnected interaction between the motor areas and epileptic networks. It has been shown that task-related changes in intracortical excitability measured by TMS increase even in healthy subjects by activating the target muscle or thinking about a movement [14], [15] and speaking [16], with changes being more prominent when studying the dominant hemisphere. This implies that exercise or thinking about exercise can, in certain patients, raise cortical excitability in a manner that is sufficient to exceed seizure threshold and precipitate an event. Patient 10 highlights this, where seizures were precipitated both by exercising as well as thinking about exercise. Furthermore, as described by Sturm et al., the stimuli involved with exercise involve emotional, motivational, and mnestic components [5]. The complex and task-specific integration of these factors involves recruitment of temporal lobe systems and provides a potential mechanism by which exercise may exert a seizure-inducing effect.

Of note, nine of the ten patients had seizures that were clearly lateralized to the left (dominant) temporal lobe. In addition to this, the two patients described by Sturm et al. also had left temporal seizures. Previous studies have shown that epileptic activity of the right temporal lobe can modulate sympathetic cardiac control [17] through activation of structures such as the insula and the amygdala. The evidence for this lateralization of heart rate regulation appears to be of greater statistical significance in males [18]. In addition, while activation of the right insular cortex was reported to produce a tachycardic response, intraoperative stimulation of the left insular cortex has conversely been shown to result in a bradycardic response [19]. As epileptic activity can modulate the autonomic nervous system, it is possible that exercise and its associated autonomic responses may provide feedback to modulate the cerebral hemispheres, preferentially activating left temporal structures given the relationship noted in this series.

Five of the patients underwent surgical treatment. However, none of these patients had successful long-term postoperative outcomes, with the seizures of all cases remaining refractory to multiple AEDs. The exact reason for this is unclear. It is recognized that seizures in TLE due to hippocampal sclerosis with a widespread epileptogenic zone are more likely to have a poorer outcome from resection [20]. It is possible that the exercise-induced seizures described have more extensive networks involved in seizure initiation and propagation, which are less likely to be completely excised by the standard resection of mesiotemporal structures.

Although regular exercise should generally be encouraged in patients with epilepsy as it may offer more of a protective effect against seizures and health promotion in general, this is not the case once a clear exercise-related trigger has been identified. In all cases in our cohort, advice was given regarding avoidance of recognized precipitants. The patients who followed this advice experienced a significant improvement in their seizure control. These included patients whose seizures were refractory to surgical resection. As mentioned, some patients would either decrease the intensity of exertion or seek alternate forms of exercise. Other patients, however, felt that the overall health benefits and enjoyment of exercise outweighed the risks from ongoing seizures and continued with their activity against medical advice. For example, Patient 6 continued to ride his bicycle, accepting the risks of ongoing seizures rather than stopping or even decreasing the intensity of his cycling.

## 4 Conclusion

For some patients, specific types of physical exertion are precipitants for temporal lobe seizures in a similar manner to other reflex epilepsies and should be routinely enquired about by the treating clinician. This may be particularly the case for patients with seizures of left temporal origin, as suggested by the lateralization noted in the patients in our series. These seizure types were also noted to be resistant to surgical resection. If a specific physical activity is identified as a trigger, an alternative form or lower intensity of exercise should be advised, with complete avoidance if this continues to cause seizures.

## Figures and Tables

**Table 1:** Characteristics of patients with exercise-induced seizures.

Patient	Sex	Age	Seizure origin	Pathology	Lateralization	EEG	MRI	PET	SPECT	Surgery	Type of exercise that induces seizures
1	F	28	TLE	HS	Right	+	+	+	+	+	Cycling
2	M	49	TLE	Cortical dysplasia	Left	−	+			−	Running
3	M	63	TLE	HS	Left	+	+			+	Running
4	F	48	TLE	Post-encephalitis	Bitemporal, left > right	+ (L > R)	−		−	−	Playing netball
5	F	50	Frontotemporal lobe	Rasmussen's encephalitis	Left	+	+			−	Dancing
6	M	74	TLE	HS	Bitemporal, left > right	+ (L > R)	+ (L)	+ (L > R)	+ (L)	−	Cycling
7	M	44	TLE	HS	Left	+	+	+		+	Weight lifting
8	F	27	TLE	Post-encephalitis	Left	+	+	+		+	Martial arts
9	M	31	TLE	HS	Left	+	−	+		+	Cycling
10	M	45	TLE	HS	Left	−	+	+		−	Running, cycling

TLE, temporal lobe epilepsy; HS, hippocampal sclerosis. Blank space denotes test not done. +, positive test; −, negative test.

## Appendix A Supplementary data

Supplementary video Exercise-induced seizure captured during video-EEG monitoring 5 min into cycling.
