# Supplementary figures and images for: Exercise-induced seizures and lateral asymmetry in patients with temporal lobe epilepsy
Source: Epilepsy Behav Case Rep. 2014 Feb 1;2:26–30. doi: 10.1016/j.ebcr.2013.12.004 (PMC4308088; doi:10.1016/j.ebcr.2013.12.004)

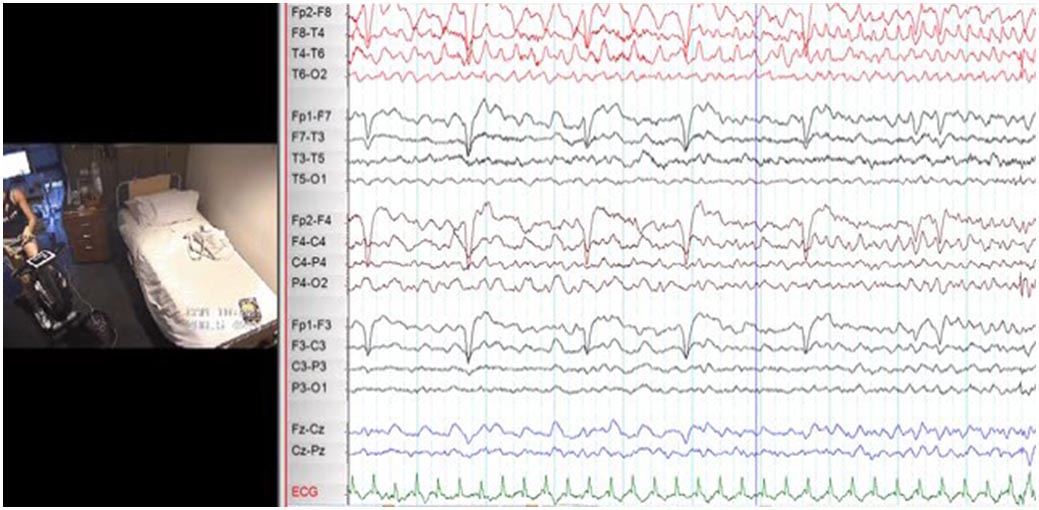

Supplement: Supplementary video — Exercise-induced seizure captured during video-EEG monitoring 5 min into cycling. [file mmc1.jpg]
